# Thermal catalytic oxidation of octachloronaphthalene over anatase TiO_2_ nanomaterial and its hypothesized mechanism

**DOI:** 10.1038/srep17800

**Published:** 2015-12-08

**Authors:** Guijin Su, Qianqian Li, Huijie Lu, Lixia Zhang, Linyan Huang, Li Yan, Minghui Zheng

**Affiliations:** 1 State Key Laboratory of Environmental Chemistry and Ecotoxicology, Research Center for Eco-Environmental Sciences, Chinese Academy of Sciences, P.O. Box 2871, Beijing 100085, China

## Abstract

As an environmentally-green technology, thermal catalytic oxidation of octachloronaphthalene (CN-75) over anatase TiO_2_ nanomaterials was investigated at 300 °C. A wide range of oxidation intermediates, which were investigated using various techniques, could be of three types: naphthalene-ring, single-benzene-ring, and completely ring-opened products. Reactive oxygen species on anatase TiO_2_ surface, such as O_2_^−•^ and O^2−^, contributed to oxidative degradation. Based on these findings, a novel oxidation degradation mechanism was proposed. The reaction at (101) surface of anatase TiO_2_ was used as a model. The naphthalene-ring oxidative products with chloronaphthols and hydroxyl-pentachloronaphthalene-dione, could be formed via attacking the carbon of naphthalene ring at one or more positions by nucleophilic O^2−^. Lateral cleavage of the naphthalene ring at different C_1_-C_10_ and C_4_-C_9_, C_1_-C_2_ and C_4_-C_9_, C_1_-C_2_ or and C_3_-C_4_ bond positions by electrophilic O_2_^−•^ could occur. This will lead to the formation of tetrachlorophenol, tetrachloro-benzoic acid, tetrachloro-phthalaldehyde, and tetrachloro-acrolein-benzoic acid, partially with further transformation into tetrachlorobenzene-dihydrodiol and tetrachloro-salicylic acid. Unexpectedly, the symmetric half section of CN-75 could be completely remained with generating the intricate oxidative intermediates characteristically containing tetrachlorobenzene structure. Complete cleavage of naphthalene ring could produce the ring-opened products, such as formic and acetic acids.

As a new type of persistent organic pollutants, polychlorinated naphthalenes (PCNs) was proposed into Annexes A and C of the Stockholm Convention (SC) on POPs in 2015^1^. There are 75 possible PCN congeners, in eight homolog groups, with one to eight chlorine atoms substituted around the planar aromatic naphthalene molecule. PCNs have been widely used in many commercial products, e.g., for wood preservation, as additives to paints and engine oils, for cable insulation, and in capacitors. Because of the structural similarities between PCNs and polychlorinated biphenyls (PCBs), PCNs are present in technical PCB formulations^2^,^3^. Yamashita *et al.*^4^ examined the concentrations and profiles of tri- through octa-chloro-substituted congeners in 18 technical PCB mixtures, and detected concentrations ranging from 5.2 to 730 μg/g. PCNs are also unintentionally generated during high-temperature industrial processes in the presence of chlorine. Of the known releases, waste incineration is considered to be the significant current source^5^, with similar formation mechanism to that of polychlorinated dibenzo-p-dioxins and dibenzofurans (PCDD/Fs)^6^. The production and use of PCNs were banned in the United States and Europe in the 1980 s, because of their toxicity and environmental persistence^7^. Nevertheless, PCNs can be released from past use, products that have not yet been disposed of, devices containing PCBs still in use, and thermal processes such as waste incineration. In accordance with the relevant SC regulations, based on a risk management evaluation and consideration of the management options, the Committee recommended a Conference of the Parties to consider listing and specifying the relevant control measures for PCNs^8^. The reduction of PCN levels is therefore a matter of public concern in the context of environmental protection.

Catalytic oxidation for the removal of chlorinated aromatic hydrocarbons has attracted much attention as a green technique^9^,^10^. TiO_2_-based catalysts are generally used for the oxidation of chlorinated aromatic compounds^11^,^12^,^13^,^14^. Lichtenberger *et al.*^12^ examined the oxidation of chlorobenzene, and 1,2-, 1,3-, and 1,4-dichlorobenzene over V_2_O_5_/TiO_2_ catalysts. A common reaction mechanism was proposed based on kinetic and *in situ* fourier transform infrared (FTIR) results. Surface phenolates are formed via nucleophilic attack at the chlorine position in the aromatic ring, followed by electrophilic substitution of the adsorbed partially dechlorinated species in the second step. Krishnamoorthy *et al.*^11^ investigated the catalytic oxidations of 1,2-dichlorobenzene over Cr_2_O_3_, V_2_O_5_, MoO_3_, Fe_2_O_3_, and Co_3_O_4_ supported on TiO_2_ and Al_2_O_3_. The TiO_2_-supported systems were more active than the corresponding Al_2_O_3_-supported ones, indicating that the support is significant in the catalytic performance of the catalyst in this reaction. Gannoun *et al.*^15^ showed that sulfated TiO_2_ nanotubes (HNTs) were a promising support for V_2_O_5_-based materials in the oxidative elimination of chlorobenzene. The formed bridged bidentate Ti and acidic sites on the HNT surface probably govern chlorobenzene oxidation and decrease the reducibility of vanadium, leading to higher reactivity at redox sites and therefore to higher-efficiency catalysts. Thus far, however, the reports to deeply identify the oxidation products and the associated mechanisms of PCNs as new POPs, are particularly scarce.

TiO_2_ is an important semiconductor material and has been used in a variety of applications such as photosplitting of water^16^, photovoltaic devices^17^, liquid solar cells, surface wettability conversion, and degradation of toxic pollutants^18^. This wide range of applications can be attributed to its nontoxicity, low cost, photostability, redox efficiency, and availability. TiO_2_ has three crystal form, i.e., brookite, anatase, and rutile. The crystal form of TiO_2_ has a decisive effect on its catalytic performance, because the electronic band gaps (EBGs) of the different forms of TiO_2_ are different. It has been reported that the photocatalytic activity of anatase TiO_2_ is limited by its small absorption range in the solar spectrum, as a result of its large EBG (*E*g = 3.2 eV). However, the larger EBG of anatase TiO_2_ has attracted great interest in its better oxidation performance. Therefore, it is of significance that the catalytic oxidation of PCNs is performed by anatase TiO_2_ with illustrating the involved deep oxidation mechanism.

In this study, the reactivity of an anatase TiO_2_ nanomaterial toward a model compound, i.e., octachloronaphthalene (CN-75), which is fully substituted with chlorine atoms, was evaluated at 300 °C. The degradation products, especially the oxidation products, were comprehensively investigated using gas chromatography–mass spectrometry (GC/MS) combined with silicane derivatization, high-performance liquid chromatography/hybrid quadrupole time-of-flight mass spectrometry (HPLC/Q-TOF-MS/MS), and ion chromatography (IC). Electron spin resonance (ESR) experiments, in combination with X-ray photoelectron spectroscopy (XPS) analysis of the TiO_2_, were used to study the role of reactive oxygen species in the degradation of CN-75. An oxidative degradation mechanism was proposed based on the findings. The results will be useful in developing methods for eliminating PCN-concentrated wastes.

## Results

### Kinetic study

**Figure 1:**
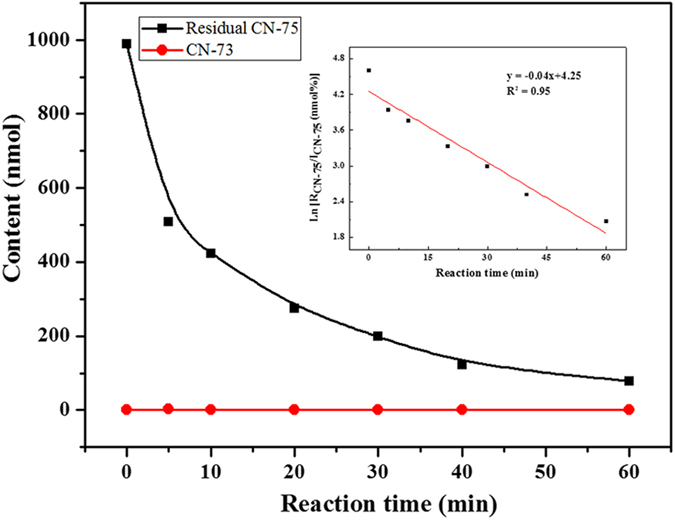
Contents of residual CN-75 and generated CN-73 as function of heating time. Inset shows pseudo-first-order kinetic plot of the reaction.

The time-dependent degradation behavior of CN-75 (990.1 nmol) over anatase TiO_2_ at 300 °C was investigated. The black squares in Fig. 1 represent changes in the amount of residual CN-75 with heating time at 300 °C, calculated based on quasi-exponential decay. The amount of CN-75 decreased from 990.1 to 78.28 nmol in 60 min. This suggests that nanosized anatase TiO_2_ is an effective catalyst for CN-75 degradation. A linear ln(*R*_CN-75_/*I*_CN-75_) versus time plot corresponding to pseudo-first-order reaction kinetics with an initial rate constant *k*_obs_ (min^−1^) of 0.04 was obtained as shown in the inset in Fig. 1(I_CN-75_ is the initial number of moles of CN-75, and R_CN-75_ is the number of moles of the remained CN-75 following heating for a given time period). It can be seen from Fig. 1 that only a small amount of 1,2,3,4,5,6,7-heptachloronaphthalene (CN-73) was detected in the hydrodechlorination products from 5 to 60 min. In contrast, in the progress of CN-75 degradation over as-prepared Fe_3_O_4_ with the similar dosage for the same reaction phases, a series of hydrodechlorination products from heptachloronaphthalenes to dichloronaphthalenes were detected^19^. The hydrodechlorination reaction of CN-75 was less favored on anatase TiO_2_ than on Fe_3_O_4_. This may be because the stability of anatase TiO_2_ is higher than that of Fe_3_O_4_, as shown by the higher EBG of anatase TiO_2_ (3.2 eV) compared with that of Fe_3_O_4_ (0.1 eV). Similarly, the weaker hydrodechlorination of decachlorobiphenyl was also found in its degradation over NiFe_2_O_4_ with EBG at 2.19 eV than over Fe_3_O_4_^20^.

### GC/MS analysis of oxidation products after derivatization

Competition between hydrodechlorination and oxidation reactions in the degradation of chlorinated benzenes over metal oxides has often been reported^9^,^12^,^20^,^21^. The reason is that lower chlorinated products and oxidation products, such as phenolate, acetate, and carbon monoxide species, have been detected simultaneously^9^,^22^,^23^. This may be explained by different types of active centers on catalysts. One of the reactions will be the main process, depending on the reaction conditions and reactants. A low level of hydrodechlorination suggests that oxidative degradation occurs preferentially. The oxidation intermediate products formed during catalytic degradation of CN-75 were studied to obtain a better understanding of the degradation pathway. Theurich *et al.*^24^ reported that 15 different oxidation intermediates were identified during the photocatalytic degradation of naphthalene in aqueous suspensions of TiO_2_ under UV irradiation. To evaluate the existence of oxidative intermediates during the reaction, the dosage of CN-75 increased from 990.1 nmol to 4,950.5 nmol. GC/MS is often used to identify unknown substances. However, the response of the oxidative degradation products often with high polarity was poor in GC/MS. Silylation is one of the derivatization procedures widely used to improve GC behavior of polar compounds containing phenolic and carboxylic groups. In this procedure, the active hydrogens could be replaced by trimethylsilyl groups, producing derivatives which are more volatile and thermally stable. Albero *et al.*^25^ reported that phenolic and carboxylic compounds in soil, such as parabens, bisphenols and triclosan, were determinated by gas chromatography tandem mass spectrometry with *in situ* derivatization of N,O-bis(trimethylsilyl)trifluoroacetamide with 1% trimethylchlorosilane (BSTFA:TMCS = 99:1, v/v). Saitta *et al.*^26^ also demonstrated 21 phenolic compounds in Italian and Turkish pistachio oil samples by means of the mass spectra of the BSTFA-TMCS derivatives. In present study, the reaction products were derivatized using BSTFA:TMCS (99:1)^27^, and then analyzed using GC/MS in EI full-scan mode. The main derivatization reactions are as follows:


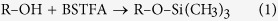



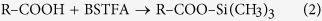


**Figure 2:**
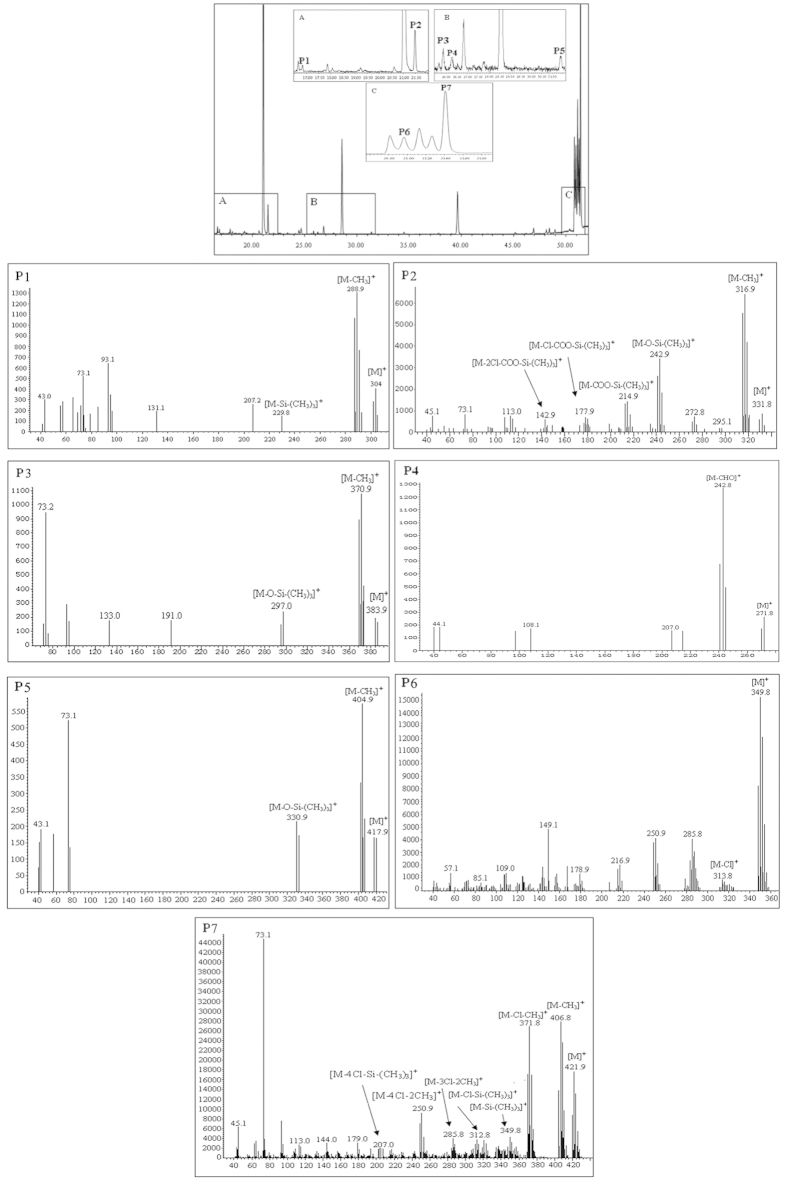
GC/MS chromatograms of derivatized products of CN-75 degradation over anatase TiO_2_ at 300 °C for 5 min.

Figure 2 shows the GC/MS chromatograms of the chemically derivatized samples after CN-75 degradation over anatase TiO_2_ at 300 °C for 5 min. Analysis of the derivatized products showed that tetrachlorophenol, tetrachlorobenzoic acid, tetrachloroacroleinbenzoic acid, tetrachlorophthalaldehyde, tetrachlorosalicylic acid, and hexachloronaphthols were produced. The list of corresponding oxidation products is given in Table 1. Full-scan MS analysis was performed to identify the structures of the detected oxidation derivatives. Qualitative analysis was performed based on the molecular ions, fragment ions, the ratio between ^35^Cl and ^37^Cl, and comparison with data in the NIST02 standard spectral database^28^. As shown in Fig. 2, clear molecular ions and fragment ions were observed for seven derivatized products. For example, the mass spectrum corresponding to Peak P2 showed the presence of derivatized tetrachlorobenzoic acid. A clear molecular ion [M]^+^ at *m*/*z* 332, and fragmentation clusters at *m*/*z* 317 [M−CH_3_]^+^, 243 [M−OSi(CH_3_)_3_]^+^, 215 [M−COOSi(CH_3_)_3_]^+^, 178 [M−ClCOOSi(CH_3_)_3_]^+^, and 143 [M−2ClCOOSi(CH_3_)_3_]^+^were observed. The isotope distributions fit a four Cl atom profile (the ratio of the peaks at *m*/*z* 332 and 334 was 1:1.3). This information clearly identifies the product as tetrachlorobenzoic acid^29^. The mass spectrum corresponding to Peak P4 showed the presence of tetrachlorophthalaldehyde^30^. The mass spectrum showed a molecular ion [M]^+^ at *m*/*z* 272 and a fragmentation cluster at *m*/*z* 243 [M−CHO]^+^. The mass spectrum of Peak P7, corresponding to the derivative of hexachloronaphthol, showed a molecular ion [M]^+^ at *m*/*z* 422 and typical fragmentation clusters at *m*/*z* 407 [M−CH_3_]^+^ and 372 [M−ClCH_3_]^+^. The identification of naphthalene rings and single benzene rings with –OH, –COOH, and –CHO substituents confirmed that oxidation reactions occurred. The presence of oxidation intermediates containing single benzene rings indicated partial splitting of the naphthalene rings during the oxidative degradation reaction. In contrast, the oxidative products only with naphthalene-ring, i.e., tetrachloronaphthols and dihydrodiol, have been determined by GC-MS during the biodegradation of 1,4-dichloronaphthalene^31^.

### HPLC/Q-TOF-MS/MS analysis of oxidation products

**Figure 3:**
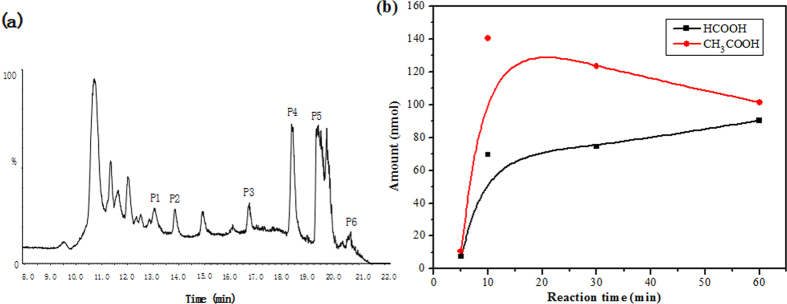
(**a**) HPLC spectrum of chemical species obtained by degradation of CN-75 over anatase TiO_2_ at 300 °C for 5 min and (**b**) distribution profiles of organic acids formed during degradation of CN-75 over anatase TiO_2_ at 300 °C.

LC/MS is a sensitive analytical technique that is widely used for the separation and quantification of highly polar products^32^,^33^. During the analytical process, polar oxidation products are efficiently ionized using the ionization techniques associated with LC/MS, enabling their identification^34^. This technique has been often applied together with GC/MS to comprehensively determine the polar species^35^. The oxidation process was therefore further investigated by monitoring the formation of oxidation intermediate products during the catalytic degradation of CN-75 over anatase TiO_2_ using HPLC/Q-TOF-MS/MS. Figure 3a shows the HPLC results for the chemical species following reaction between CN-75 (4,950.5 nmol) and anatase TiO_2_ (50 mg) at 300 °C for 5 min. Tetra-chlorophenols, tetrachlorobenzenedihydrodiol, hydroxypentachloronaphthalenedione (OH-PeCN-dione), hydroxypentachloronaphthalene (OH-PeCN), and hydroxyhexachloronaphthalene (OH-HxCN) were determined as degradation products (Table 2). However, the isomer patterns of the hydroxyl congeners could not be identified because of limitations associated with the external standards. These results further show that oxidation intermediates with naphthalene rings and single benzene rings were produced during the oxidative degradation reaction. However, the only hydroxyl-oxidative products with naphthalene ring, i.e. hydroxyl-trichloronaphthalene (TrCN), -tetrachloronaphthalene (TeCN), -PeCN, and -HxCN have been determined by HPLC/Q-TOF-MS/MS during the degradation of CN-75 on Fe_3_O_4_^19^. This suggests the occurrence of deep oxidative degradation of CN-75 on anatase-type TiO_2_.

### Analysis of oxidation products by IC

Literature reports have indicated that chlorinated aromatic compounds containing hydroxyl, aldehyde, and carboxyl groups can be easily ring-cracked to smaller organic molecules such as formate and acetate^11^. Ma *et al.*^36^ detected the formation of surface formate species using *in situ* FTIR spectroscopy in low-temperature 1,2-dichlorobenzene oxidation over water-resistant Fe–Ca–O_*x*_/TiO_2_ catalysts. Similar results were reported for the catalytic oxidation of 1,2-dichlorobenzene over Ca-doped FeO_*x*_ hollow microspheres^37^. Formic, acetic, and propanoic acids have been detected during degradation of decachlorobiphenyl over Fe_3_O_4_^19^. In the current study, ring-cracked products were detected, using IC, in the reaction between CN-75 (990.1 nmol) and anatase TiO_2_ (50 mg) at 300 °C. Formic and acetic acids were the main ring-cracked degradation products, as shown in Fig. 3b. The amount of acetic acid rapidly increased to a maximum of 140.6 nmol after heating for about 10 min, and then decreased with heating time. In contrast, the formic acid content increased steadily with heating time, with a maximum content of 90.4 nmol at 60 min. These oxidation products indicate that TiO_2_ also facilitates the ring-cracking oxidation pathway of chlorinated aromatics.

## Discussion

**Figure 4:**
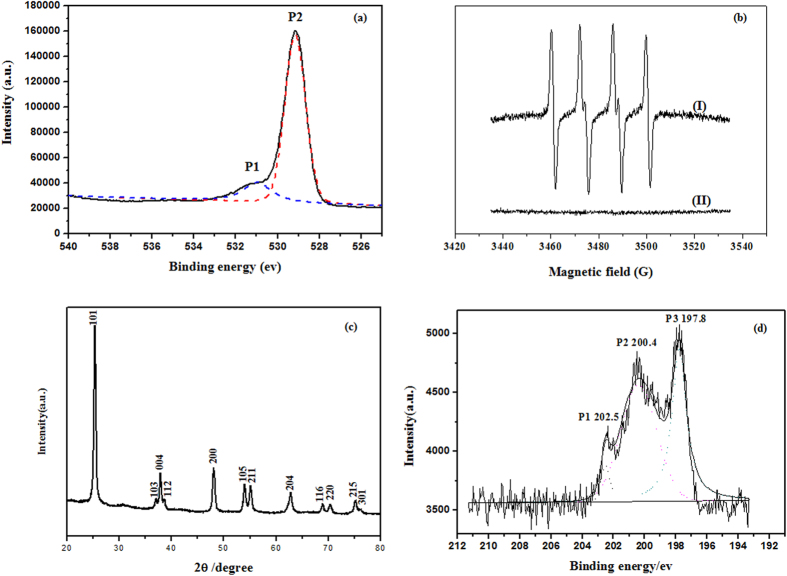
(**a**) O 1s XPS spectrum of TiO_2_ catalyst, (**b**) ESR spectra of O_2_^−•^ (I) and •OH (II) generated by reaction of anatase TiO_2_ and CN-75 at 300 °C for 10 min, (**c**) XRD pattern of TiO_2_ catalyst and (**d**) Cl 2p XPS spectrum of TiO_2_ sample after the reaction for 10 min.

The presence of active oxygen species on nanosized anatase TiO_2_ catalysts is believed to contribute to the occurrence of oxidation reactions during CN-75 degradation^19^. The O 1s XP spectrum of the anatase TiO_2_ catalyst is shown in Fig. 4a. The peak at 530.97 eV (denoted by P1) is attributed to surface oxygen and adsorbed oxygen species, and the peak located at 529.15 eV (denoted by P2) is attributed to lattice oxygen^38^. Similar oxygen species were detected on the surface of Ca-doped FeO_*x*_ hollow microspheres and CaCO_3_/α-Fe_2_O_3_ composite catalysts^37^. A high proportion of surface oxygen on the metal oxide catalyst increases the activity in low-temperature oxidative degradation of 1,2-dichlorobenzene.

Reactive oxygen species such as O_2_^−•^ and •OH are strong electrophilic oxidants. They can attack organic substrates, leading to their degradation and ultimately to their total mineralization to CO_2_ and H_2_O^39^,^40^. Its role in a range of photocatalytic oxidative degradation reactions, including those of pathogenic bacteria over NiO/SrBi_2_O_4_^41^, rhodamine B over TiO_2_^42^, and azo dyes over Ag/AgBr/TiO^43^, have been confirmed by ESR spectroscopy. ESR spectroscopy, with DMPO as the spin-trapping agent, was used to obtain information on the active radicals involved, to determine whether O_2_^−•^ and •OH were available products in the decomposition of CN-75 over anatase TiO_2_. A reaction was performed between anatase TiO_2_ (50 mg) and CN-75 (990.1 nmol) at 300 °C for 10 min. The reaction products were immediately dissolved in dimethyl sulfoxide (DMSO), and then characterized using an ESR analyzer, as shown in Fig. 4b. Four peaks were observed, and the hyperfine constants, i.e., *α*_N_ = 12.7429 G, *α*_H_ = 10.0304 G, and *g* = 2.0103, coincided with those previously reported for DMPO–O_2_^−•^ (Fig. 4b-I)^9^. The results identify that the superoxide anion may be involved in CN-75 degradation, resulting in the formation of a series of oxidation products and perhaps even into formic acid and acetic acid. The DMPO–∙OH species were examined under identical conditions, except water was used as the solvent instead of DMSO. No obvious signal was observed, as shown in Fig. 4b-II. This differs from the photocatalytic degradation of many organic molecules, in which ∙OH species are often identified^41^,^42^,^43^.

**Figure 5:**
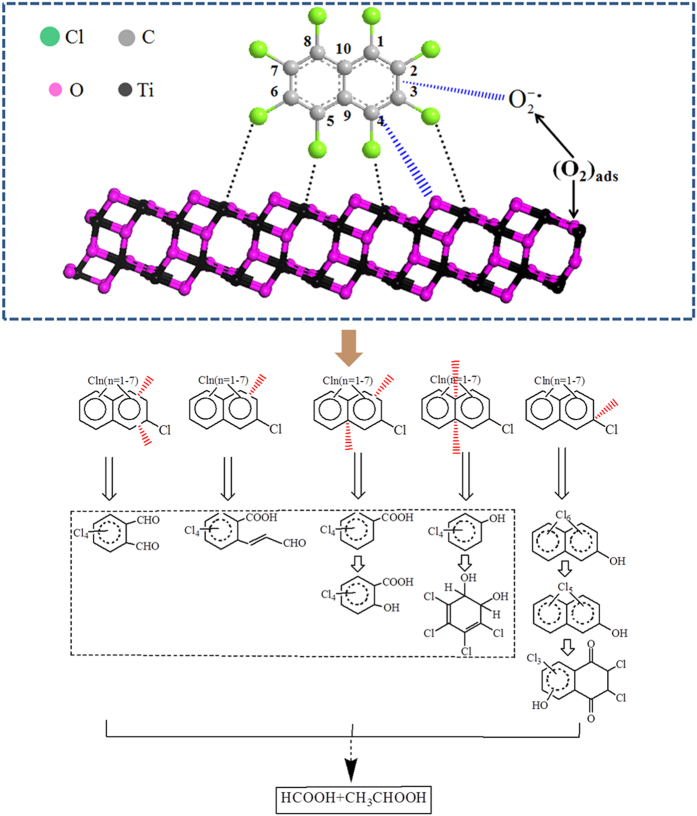
Possible degradation pathways of CN-75 over anatase TiO_2_.

An oxidative degradation pathway (Fig. 5) is proposed, based on the available oxygen species and the detected oxidation intermediates. The (101) surface is the most stable and frequent surface of anatase TiO_2_, as shown in Fig. 4c, which was therefore selectively took as a model^44^,^45^,^46^. It has the same periodicity as the bulk truncated surface and exposes undercoordinated pentacoordinated Ti cations (Ti_5c_) and dicoordinated oxygen anions (O_2c_), and fully coordinated Ti_6c_ cations and tricoordinated oxygen anions (O_3c_)^47^. Coordination theory states that unsaturated ions are prone to bond with ligands^23^. It is therefore hypothesized that CN-75 molecules are adsorbed on the anatase TiO_2_ surface via coordination interactions between Lewis acid Ti_5c_ cations and Lewis base Cl^21^. When CN-75 degraded on the surface of the anatase TiO_2_ catalyst, firstly, dissociative adsorption of CN-75 on the central Ti_5c_ cations occurs, followed by the attack of carbon atom potential to accepting the electrons by reactive nucleophilic oxygen O^2−^ species. This results in C–Cl bond cleavage and subsequent Ti–Cl bond formation. Association of the free chloride ions with Lewis acid Ti ions occurs during CN-75 degradation over anatase TiO_2_. This is confirmed by the Cl 2p core-level XP spectrum of the catalyst after heating for 10 min (Fig. 4d). Three peaks (denoted by P1, P2, and P3) are observed. The peak at 197.8 eV corresponds to Cl bonded to Ti^4+^, with a net charge of −1, indicating possible formation of TiCl_4_ during degradation of CN-75^48^. In this reaction pathway, OH-HxCN and OH-PeCN can be formed via nucleophilic attack by basic O^2−^. Further nucleophilic attack can occur at other positions on PCNs, forming species such as OH-PeCN-dione. The formation of naphthol species was detected during photocatalytic degradation of naphthalene over TiO_2_^24^.

Superoxide O_2_^−•^ species are electrophilic. They have been reported to be formed by transformation of adsorbed O_2_ molecules^19^,^49^. When a subsurface oxygen vacancy is present, it is energetically favorable for O_2_ to adsorb at a Ti_5c_ site close to this defect. On adsorption, the extra charge associated with the defect is transferred to the O_2_ molecule, converting it to a superoxide O_2_^−•^ species. The strongly reactive electrophilic O_2_^−•^ species can attack the π-electron cloud of the naphthalene ring, which has a highly dense electron population. This leads to the cracking of the naphthalene ring at different positions. The detection of tetrachlorophenol and the resultant tetrachlorobenzenedihydrodiol indicates that one of the rings in the naphthalene ring of CN-75 is first opened through C_1_–C_10_ and C_4_–C_9_ bond cleavage. Breakage of the C_1_–C_2_ and C_4_–C_9_ bonds in one ring could result in the formation of tetrachlorobenzoic acid, which is further oxidized to tetrachlorosalicylic acid. Similarly, the breakage of C_1_–C_2_ or and C_3_–C_4_ bonds could lead to the formation of tetrachloroacroleinbenzoic acid or and tetrachlorophthalaldehyde, respectively. These results show that lateral cleavage of one naphthalene ring at different C–C bond positions by electrophilic O_2_^−•^ could occur, leading to formation of various single-benzene-ring oxidation products. Unexpectedly, the symmetric half section of CN-75 could be retained along with generation of complex oxidation products containing the tetrachlorobenzene structure.

It is important to note that the reaction pathways via electrophilic and nucleophilic attack by reactive oxygen species such as O^2−^ and O_2_^−•^ are not independent of each other. The newly formed chlorinated naphthol species can also be attacked by reactive oxygen species such as O_2_^−•^. Moreover, oxidation products with both naphthalene and single benzene rings can be further attacked by reactive oxygen species, and completely cracked to small molecules such as formic and acetic acids. A wide range of oxidation products such as naphthols, phenols, hydroxy-diones, benzoic acids, acroleinbenzoic acid, phthalaldehyde, salicylic acid, dihydrodiols, and formic and acetic acids, with chlorinated naphthalene or benzene rings, or without aromatic rings, were detected during the degradation of CN-75 over anatase TiO_2_. This is different from the previously reported results for CN-75 degradation over Fe_3_O_4_ micro/nanomaterials^19^, in which only chloronaphthol species, and formic acid and acetic acids were detected as the oxidation products under the same experimental conditions. This shows that oxidative degradation of CN-75 on anatase TiO_2_ was more extensive that on Fe_3_O_4_ micro/nanomaterials. Deep oxidative degradation of CN-75 on anatase TiO_2_ occurs possibly because of the electronic structure with an EBG of 3.2 eV and the reactive oxygen species on its surface.

## Methods

### Chemical reagents

Anatase TiO_2_ (nanopowder, diameter <25 nm) was supplied by Sigma-Aldrich (USA). CN-75 (Supelco, USA) was laboratory analytical grade and used without further purification. HPLC-grade ethyl acetate was purchased from Fisher Scientific (Geel, Belgium). Chromatographic-grade methanol, acetonitrile, and hexane were purchased from Dika Technologies (Lake Forest, CA, USA). Derivatization reagents, BSTFA:TMCS = 99:1 were supplied by Supelco (USA).

### Degradation experiments

Degradation experiments were performed in sealed glass ampoules. Prior to the reaction, a hexane solution of CN-75 (990.1 or 4,950.5 nmol) was injected into an ampoule and subsequently evaporated to dryness at room temperature, and then mixed with later added 50 mg of anatase TiO_2_. The samples were heated at 300 °C for an appropriate time. A blank experiment was performed in the absence of TiO_2_ under the same conditions. All experiments were performed in triplicate to ensure repeatability of the results.

### Degradation product analysis

After the decomposition reaction, the ampoule was cooled to room temperature and crushed, and the sample was extracted. The unreacted CN-75 and newly formed PCNs were analyzed using an Agilent 6890 gas chromatograph equipped with a DB-5 MS capillary column (30 m × 0.25 mm i.d., 0.25 μm film thickness) and an Agilent 5973 N mass selective detector. Helium (≥99.999%) at a flow rate of 1 mL/min was used as the carrier gas, and the injector was set at 260 °C. The column temperature was set at 75 °C for 2 min, gradually increased to 150 °C at 20 °C/min, then increased to 205 °C at 1.5 °C/min, and finally increased to 270 °C at 2.5 °C/min. The diluted sample (1.0 μL) was injected in split-less mode. An electron ionization system with an ionization energy of 70 eV was used.

For oxidation product analysis, the reaction products obtained after CN-75 degradation over anatase TiO_2_ at 300 °C for 5 min were extracted by the mixture solvent of hexane/ methanol/ ethyl acetate (1:1:1, v/v/v). The extract was dehydrated using a column packed with anhydrous sodium sulfate, and then evaporated under stream of nitrogen to dryness. Dry residue was dissolved in 0.2 mL of derivatizing reagent BSTFA:TMCS (99:1) and vortexed. The mixture reacted at room temperature for 60 min, and the derivatization products were analyzed using GC/MS. The column temperature was initially 50 °C, and increased to 180 °C (for 2 min) at 10 °C/min, to 210 °C at 1 °C/min, and to 280 °C at 10 °C/min. The carrier gas was helium at a flow rate of 1 mL/min.

The oxidation products were also analyzed using HPLC/Q-TOF-MS/MS (Micromass Q-TOF micro, Waters, USA). After the degradation of CN-75 (4,950.5 nmol) over anatase TiO_2_, product samples were extracted using HPLC-grade methanol, filtered through a 0.45 μm mesh membrane, and concentrated to approximately 100 μL. The oxidation products were detected using a Supelcosiltmlc-18 C18 column (Sigma; 4.6 mm × 250 mm; 5 μm particle size). The elution flow rate was 0.5 mL/min with a gradient of 0.1% acetic acid in water–acetonitrile [acetonitrile concentrations 0% (isocratic, 5 min), 70% (isocratic, 5 min), 70–90% (linear, 5 min), 90–100% (linear, 5 min), 100% (isocratic, 5 min), and 0% (isocratic, 4 min)]. MS was performed using a Waters Micromass Quattro Premier XE (triple-quadrupole) detector, equipped with an electrospray ionization (EI) source (Micromass, USA). The mass analyzer was operated in negative ionization (EI^−^) mode and the optimized parameters were source temperature 120 °C, desolvation temperature 200 °C, capillary voltage 2.50 kV, desolvation gas flow rate 600 L/h, and cone gas flow rate 50 L/h.

The organic acid oxidation products such as acetic and formic acid were analyzed using IC. The degradation samples obtained from the reaction of CN-75 (990.1 nmol) and anatase TiO_2_ (50 mg) were extracted three times with 15 ml deionized water for 10 min each time under ultrasonication. And then the combined extracts were filtered through a 0.45 μm mesh membrane for IC measurements. The employed IC was a DIONEX AS 5000 instrument equipped with an AS-AP automated sampler. A Dionex AS11-HC guard column (50 × 4 mm i.d.) and a Dionex AS11-HC analytical column (250 × 4 mm i.d.) were used for the analyses. The analyses were performed at 30 °C with a potassium hydroxide eluent that was generated from a Dionex EG on line and run with a linear gradient at a flow rate of 1.0 mL min^−1^.

### XPS and ESR

The surface element oxidation states of the TiO_2_ catalyst, which reacted with CN-75 at 300 °C for 10 min, were investigated using XPS (Escalab 250), with monochromated Al Kα (1,486.6 eV) radiation (200 W, 200 eV) as the X-ray source. The operating pressure was ~1 × 10^−8^ Torr.

The radical species formed during degradation were investigated using ESR spectroscopy (ESP 300 E electron paramagnetic resonance spectrometer, Bruker) with 5,5-dimethyl-1-pyrroline *N*-oxide (DMPO; Sigma Chemical Co.) as the spin-trapping agent. Typically, anatase TiO_2_ (50 mg) and CN-75 (990.1 nmol) reacted at 300 °C for 10 min. A reaction using anatase TiO_2_ but without CN-75 was also examined under the same conditions for comparison. The settings for the ESR spectrometer were center field, 3,485 G; sweep width, 100.0 G; microwave frequency, 9.8 GHz; and power, 10 mW.

## Additional Information

**How to cite this article**: Su, G. *et al.* Thermal catalytic oxidation of octachloronaphthalene over anatase TiO_2_ nanomaterial and its hypothesized mechanism. *Sci. Rep.* **5**, 17800; doi: 10.1038/srep17800 (2015).

## Figures and Tables

**Table 1:**
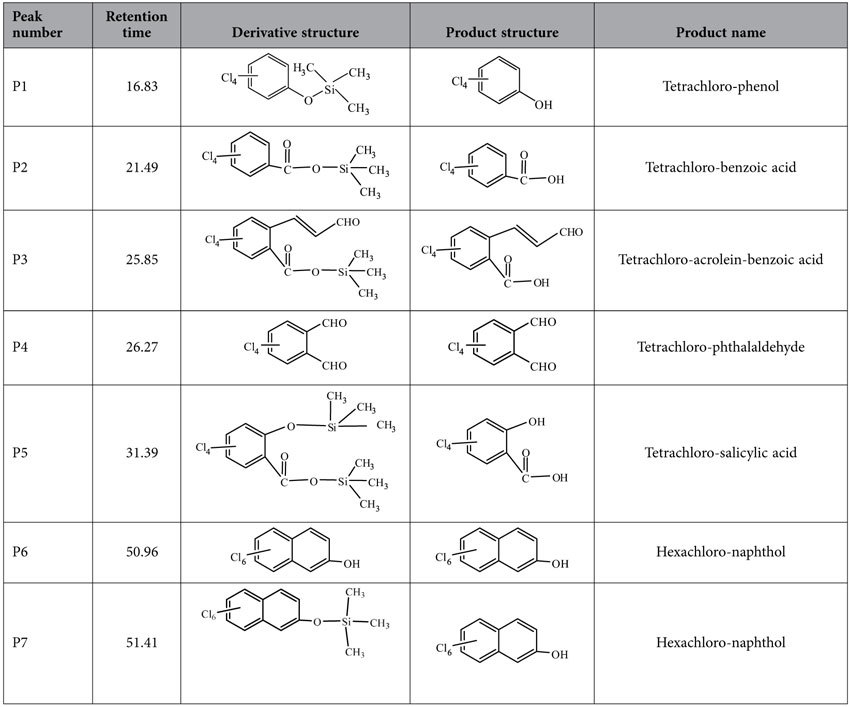
Oxidative products following degradation of CN-75 over anatase TiO_2_ at 300 °C for 5 min, determined by GC/MS after the derivatization.

**Table 2:**
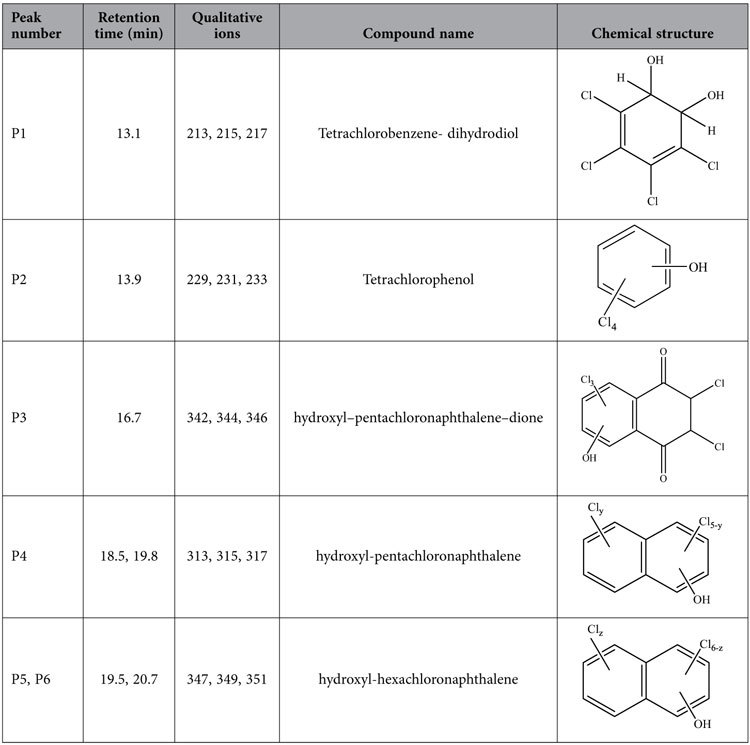
Oxidative products following degradation of CN-75 over anatase TiO_2_ at 300 °C for 5 min, determined by HPLC/Q-TOF-MS/MS.
